# Cripping auto-/ethnography?

**DOI:** 10.3389/fsoc.2025.1577749

**Published:** 2025-05-21

**Authors:** Yvonne Wechuli

**Affiliations:** Unit Disability, Inclusion and Social Participation, Institute of Social Work and Social Welfare, Faculty of Human Sciences, University of Kassel, Kassel, Germany

**Keywords:** cripping, crisis of representation, ethnography, autoethnography, disability studies, ableism in academia, ableism, illness narratives

## Abstract

Given the crisis of representation (of *the other*) and complicated histories of othering, ethnography seems to be a methodology in need of cripping. Autoethnography, then, is one approach to solve said crisis of representation. Down to classics like Robert Murphy's *The Body Silent*, Disability Studies often use authors' autobiographical experience in a way that may be called autoethnographic. However, Disability Study's authors rarely engage with methodological literature on autoethnography. Moreover, autoethnographic literature frames *The Body Silent* and others as first-person illness narratives, which I read as one indication that autoethnography might play into a *tragedy* narrative of disability. This paper tries to think through what it can mean to crip auto-/ethnography. To this end, I introduce cripping as an emancipatory strategy that promotes changing how one feels about disability and gather previous attempts of cripping academic knowledge production, which specifically center ableist temporal and emotional norms. In a second step, I outline ethnography and autoethnography as methodologies of interest and elaborate, which methodological development could be harnessed for cripping and in which ways both could benefit from further cripping.

## 1 Introduction

This conceptual analysis asks the question what it would mean to crip ethnography and/or autoethnography. Cripping is an emancipatory strategy discussed in research and activism whose proponents call for a re-evaluation of one's feelings regarding disability toward the affirmative (McRuer, 2006). I explore the possibilities of a cripped auto/ethnography here based on the design for a planned post-doc project on affective resistance to accessible open space planning. Within Disability Studies the emerging sub-field of “critical access studies” (Hamraie, 2017, p. 13) investigates why (demands for) accessibility fail to realize an inclusive society via architectural and technological design. Several authors discuss affective resistance to accessible design (Siebers, 2009; Titchkosky, 2011; Fritsch, 2013) and some authors specifically report on affective resistance to an accessible design of open spaces as well (Clare, 2015; Kafer, 2017).

As I have argued elsewhere (Wechuli, 2022), writing about affect and emotion in Disability Studies can focus different aspects. One of those aspects are affective reactions to disability, which tend to be discussed as socio-culturally shaped projections yet remain undertheorized to date.^1^ Knowledge production here is usually based on observations of the strange behavior of able-bodyminded people toward disabled people, which Disability Studies explore either autoethnographically or on the basis of qualitative data. In a second step, authors apply psychoanalytical, sociological or philosophical theories to reflect on the assumed emotional foundations of this strange behavior (Wechuli, 2024). Often, affective reactions are condensed into one single, distinct emotion concept (Scheve and Slaby, 2019), e.g. when Hughes (2012, p. 68) reconstructs fear, pity, and disgust as “the major—though not the exclusive—building blocks of the emotional infrastructure of ableism”.

To justify an empirical approach to affect, I draw on Sauerborn and Albrecht's (2024) understanding of affectivity for the social sciences that differs from a concept of affect that is partly common in cultural studies, which frames affect as a phenomenon that cannot be grasped in language at all. They identify three characteristics of affectivity that enable empirical research to this elusive phenomenon, namely that it can be observed, narrated and experienced. That affectivity is observable suggests ethnography as a methodology whereas autoethnography seems fitting to capture experience (Sauerborn and Albrecht, 2024).

In the following, I will introduce cripping as an emancipatory strategy (Section 2) and share previous reflections on a cripped knowledge production from Disability Studies (Section 2.1). Then, I will give an overview of ethnography (Section 3) and autoethnography (Section 4) and elaborate entry points to crip these methodologies as well as central critiques. Lastly, the potentials and pitfalls of cripping auto-/ethnography are discussed in conclusion (Section 5).

## 2 Cripping

“Cripping” (Sandahl, 2003) stands in the tradition of older calls for an affirmative re-evaluation of disability as a source of pride (see e.g. Corbett, 1994; Campbell, 2009; Clare, 2015). The pejorative term *crip* (*cripple*) emerged from activist contexts where the term has been reappropriated despite, or perhaps because of, its history of pejorative use (Johnson and McRuer, 2014b). “[W]ords to shock, words to infuse with pride and self-love, words to resist internalized hatred, words to help forge a politics” (Clare, 2015, p. 84). A reference to *crip* thus proclaims pride by accepting the ascribed social identity without accepting the associated devaluation (Clare, 2015). Reappropriated pejorative terms can draw attention to shared hurt feelings and marginalization and at the same time cause deliberate irritation (Mingus, 2011). Despite the associated hopes of gaining allies for a political agenda, the recycling of terms infused with negative associations remains a complex process (Liddiard and Slater, 2018)—an emotionally complex process as Alison Kafer (2021, 415; her italics) elaborates:

“I remain deeply attached to *crip*—as a word, an orientation, an affiliation, a feeling. […] And yet, the fact that I love the feel of the word across my skin, the sound of it on your tongue, doesn't change the fact that the word has edges and edges bind”.

Cripping is often used as a verb, for instance in the description of this research topic, which calls for—among other aspects—cripping research methods, research practices and modes of analysis, in the same way that several authors in disability studies have argued for a cripping of professional standards such as the rules of academic knowledge production (see Section 2.1). Beyond academia, one can seek to crip different areas of life such as sexuality and intimacy (Liddiard, 2018) or family life (Goodley and McLaughlin, 2008; Goodley and Runswick-Cole, 2013) or aspects of disability experiences such as pain (Sheppard, 2020b), or even physical concepts such as time (Samuels, 2017; Kafer, 2021).

Cripping aims to strategically reorient one's (emotional) associations with disability in the sense of “ways of knowing and feeling disability” (Parrey, 2020, p. 37) and learning to feel *differently* about disability (Corbett, 1994). How do disabled people and their allies achieve this reorientation? In order to learn to feel differently about disability, cripping invites the celebration of disability as difference—specifically by re-evaluating even seemingly negative aspects of disability experiences as spaces of possibility. For example, pain can be affirmed as constitutive of being alive (Mintz, 2011), as offering an occasion to focus one's attention or to take a break (Scheuer, 2011). People living with chronic pain may find pleasure in inactivity (Sheppard, 2020a) or new temporal norms (Gould, 2017). In general, many scholars and activists particularly value the potential of Disability Arts to convey an affirmative image of disability (Siebers, 2009). Moreover, Disability Studies make disability a majority issue by framing able-bodymindedness as only ever temporary (Zola, 1993; Davis, 2002). “Unless we die suddenly, we are all disabled eventually. Most of us will live part of our lives with bodies that hurt, that move with difficulty or not at all” (Wendell, 1989, p. 108). Cripping not only promotes an affirmative re-evaluation of disability but also provides a rationale why one should feel proud of disability, namely due to disability's potential to subvert norms. This subversive potential has been formulated in detail for norms around interconnectedness and desire (Wechuli, 2022).

As I have argued elsewhere (Wechuli, 2022), cripping as a strategy entails certain benefits but also costs that include emotional costs. In general, affirmative reappraisals counteract *tragic* notions of disability and promise solidarity. Advocates of cripping describe the expected benefits of this strategy as a radical transformation in the sense of collectively imagining otherwise (Anzalduá, 2012). Affirmative re-evaluations of disability are justified here by the fact that disability has the potential to subvert compulsory able-mindedness (McRuer, 2006) as Liddiard (2018, p. 37–38) explains with regard to a cripped sexuality: “Assimilation is never the goal; ‘passing'—performing normal—is counterintuitive. Crip doesn't seek to normalize or individualize disability or desire, but seeks to draw upon and center its very queerness as a moment of reflection”.

These endeavors are based on an idea that had already emerged in the discourse around Disability Pride, namely to use the lived experiences of disabled people to formulate emancipatory values and norms (Longmore, 1995).

“Beyond proclamations of pride, deaf and disabled people have been uncovering or formulating sets of alternative values derived from within the deaf and disabled experiences. […] That analysis needs to be made not just because majority values are impossible for people with disabilities to match up to, but more importantly, because they have proven destructive for everyone, disabled and nondisabled alike” (Longmore, 1995, p. n.p.).

Thus, cripping seeks to generate emancipatory knowledge—for everyone—based on disabled people's lived experiences by questioning ableist ideals such as beauty, independence, individual achievement or self-control (Goodley, 2014). Interdependence, interpersonal connection and community are examples of alternative values as mentioned by Longmore (1995), which are intended to replace unattainable ideals (see also Goodley, 2014).

As a strategy, cripping is also associated with certain costs. Most prominently, cripping has been understood as elitist endeavor at the expense of disabled communities. Older disabled people, who make up the majority of disabled people in (aging) industrialized nations—just as the concept of *temporary* able-bodymindedness suggests (Zola, 1993; Davis, 2002)—rarely identify as crip. “Approximately half the people affected by disability are older people, and they are less likely to identify as disabled, let alone to deploy the term ‘crip'; for them, illness and impairment are naturalized as part of getting older” (Arciul and Shakespeare, 2023, p. 26). Such allegations of being unsolidary (Wechuli, in print) were prominently brought forward against the notion of “cripistemologies” (Johnson and McRuer, 2014a)—a neologism that combines *crip* with *epistemology*. Despite the authors' intention to draw attention to exclusion, their avant-garde terminology was read as an exclusionary, fashionable yet inaccessible term (Johnson and McRuer, 2014b).

Moreover, proud affirmations do not feel equally available across all embodiments, experiences and etiologies of disability (Clare, 2015; Price, 2015; Kafer, 2021). Disabled people who struggle with the impairment effects (Thomas, 1999) describe that it can feel almost impossible cultivate pride in disability. “I am not entirely sure I could ever wear a Proud-To-Be-Disabled T-shirt” (Meekosha, 2000, p. 814). Any celebration of a crip coming-out implies a sense of choice and control over one's embodied experience that unreliable bodyminds may not grant. Involuntary disclosures of one's disability status instead trigger feelings of shame and fear, which make it difficult to feel only or even *simply* proud in relation to disability (LaCom, 2007). Pain, as another example, complicates the (vague) demand to desire disability (Price, 2015) as does any traumatic history of impairment acquisition respectively the recognition that some ways of becoming disabled are unjust. “What comes after trauma? Can crip? Or does crip as radicalized stance, as community affiliation, feel less available, less useful, less hopeful to those disabled through violence?” (Kafer, 2021, p. 423). Similarly, theorizations of the subversive potential of crip time may differ from lived experiences (Kafer, 2021; see also Samuels, 2017). “[A]ctually inhabiting such temporalities may not feel good; theorizing the transgressive possibilities of crip time and living in crip time may bring different affective responses” (Kafer, 2021, p. 429).

Cripping as an emancipatory strategy unfolds a tension between subjective and political needs, possibilities and consequences. As feelings cannot be changed at will, expectations of pride can have an exclusionary effect (Schmechel, 2022). “Body politics or queer politics are always politics of emotion as they are about who has the right to feel certain feelings, and which feelings are required in order to belong to a certain community” (Schmechel, 2022, p. 155). At once, even critics do not deny the political significance of a deliberate emotional re-evaluation of disability toward the affirmative for socio-cultural change (Watermeyer, 2009). A more inclusive understanding should at least acknowledge that feeling proud of disability may be difficult to achieve (Campbell, 2009; Clare, 2015; Sheppard, 2020a) and may be complicated by lived experiences such as pain or violent and socially unjust etiologies. Furthermore, a more accessible approach to cripping should practice a continuous rethinking of its terminology.

### 2.1 Cripping academic knowledge production

Cripping has the potential to initiate reforms in knowledge production, as it may challenge ableism in academia. In the following, I outline what proponents of cripping have previously written about ways to crip research. Centrally, they argue to attend to crip time, one's own emotionality and—related to both—self-care.

Ableist academic orientations are intimately related to temporal norms—a normalization of overwork, and a culture of perfectionism rather interested in the end-product than the work process (Leigh and Brown, 2020). Consequently, claims for “crip time” (Kafer, 2013, p. 25) hold substantial subversive potential (Kafer, 2013, 2021; Bê and Sheppard, 2023; Sheppard, 2020b; Samuels, 2017) in academic knowledge production. “[T]heories of crip time also highlight how people are refusing and resisting those very expectations, thereby creating new affective relations and orientations to time, temporality, and pasts/presents/futures” (Kafer, 2021, p. 428). Denouncing normative time frames—either deliberately or based on one's needs e.g., to take time for breaks seriously—can promote wellbeing, a pleasurable engagement with one's body or consciousness for the present tense (Samuels, 2017; Sheppard, 2020b; Liddiard et al., 2019). As editors of a special issue on representations of chronic illness in Disability Studies, Bê and Sheppard (2023) denounced normative time frames in the publishing process for the sake of crip time by considering potential phases of sick leave from the onset.

“We sought ways to make our practice as academics inclusive, while acknowledging that we are ourselves restricted by the structures imposed on us by academic institutions; a part of that was making time to *be ill*, to acknowledge that those times would not necessarily be predictable” (Bê and Sheppard, 2023, p. 137; their italics).

Similarly, Liddiard and Watts (2022) report on a participatory research project that rethought normative schedules for qualitative research in order to make time for self-care. In their experience, this changed approach to temporality greatly increased accessibility for young disabled co-researchers.

“I feel working in this way has enabled me to contribute more to the project as I've been able to do it when I feel well enough rather than forcing myself to do something when my mind and body are screaming no. This has kept my love and enthusiasm for the project high” (Liddiard and Watts, 2022, p. 40).

Johnson and McRuer (2014a) frames a prioritization of her wellbeing and self-care over work and family commitments rather as act of crip wilfulness (Ahmed, 2010) than in temporal terms, yet describes similar outcomes.

“I am not un*able* to travel; I am frequently un*willing*. The inter-implications of capacity and debility have led me to this place of crip willfulness, which sounds like a mean place of stubborn resistance, but feels like a calm relinquishing of fantasies that I can force things (situations, bodies, emotions, sensations) to be other than they are. It is a refusal to insist—a refusal to act in accordance with the system of compulsory able-bodiedness—that requires individuals to mask, suppress, and disregard discomfort in the process of determining what is possible, of what we are capable” (Johnson and McRuer, 2014a, p. 136; their italics).

Johnson and McRuer (2014a) describe an academic knowledge production that prioritizes self-care and mutual care over competitive orientations toward sensational research results as “cripistemologies”^2^ in the sense of cripped epistemologies—“[t]he tension between a long-standing cripistemological yearning to attend patiently, carefully, and collectively to varied sensations, on one side, and, on the other, the neoliberal compulsion to get better and to be better/sensational/exceptional…” (Johnson and McRuer, 2014a, p. 138).

Besides an application of crip time in academia, several authors discuss the place of emotionality in research. Price and Kerschbaum (2016, p. 33) challenge expectations of emotional detachment in qualitative methodologies: “Why does so much qualitative-methodology literature give the impression of emotional calm on the part of the researcher?” They argue that emotional involvement based on researchers' own experiences may contribute to deepen understanding—in their specific example on the importance of accessibility. Qualitative methodologies can, thus, gain from considering disability as an integral rather than a disruptive factor from the beginning of the research process. Stephanie Kerschbaum reports not only an ease to conduct and analyze interviews but also an intense emotional, joyful reaction to this ease facilitated by a use of sign language.

Similarly, one can state ableist expectations of emotional detachment in scientific presentations (Donaldson and Prendergast, 2011; Gunaratnam, 2021)—even though presentations and their preparation are a common source of anxiety (Gunaratnam, 2021). In their editorial to a special issue of the *Journal of Literary and Cultural Disability Studies* entitled “There's no crying in Disability Studies”, Donaldson and Prendergast (2011) reflect on their joint experience of breaking such expectations by crying during their conference presentations.

“Emotion and the expression of emotion are also gendered in significant ways. Tears are feminine, and hence trivialized. Crying during a conference presentation is in one respect a failure to regulate the emotions. It signifies a moment of vulnerability that threatens to undermine the authority of the speaker and, further, in this particular case, it appears to resuscitate the pity narrative that undermines disability rights. On the other hand, crying at a conference presentation is a transgression that foregrounds issues central to both feminism and Disability Studies in potentially productive ways. Our bodies, and our minds, do not always conform to prescribed norms and regulations. Crying when one wishes not to cry is both a bodily refusal and an inability to contain or to be contained by these rules” (Donaldson and Prendergast, 2011, p. 130).

Expectations of an emotionally detached presentation style ultimately reproduce a binarization that positions researchers as able-minded—even in research on mental health. Beyond emotional detachment, there are many unwritten conventions in academic conferences as pointed out by neurodiverse presenters who feel pressured to minimize their difference. Such conventions—how to present, how to ask questions, how to respond to (challenging) comments, how to socialize—can make conferences inaccessible (Gunaratnam, 2021).

To crip ableist presentation styles may translate to asking how a practice of *vulnerable* presentation beyond self-control could look, sound and feel like—a performance that breaks with the expectation of an implicitly able-minded presentation (Gunaratnam, 2021). Price and Kerschbaum (2016) read emotional engagement and familiarity with inaccessibility as motivation to make interview settings as accessible and, thus, pleasant as possible for their interviewees. The same could be said for academic conferences—or even the classroom (Fritsch, 2024)—where one can learn from the lived experiences of disabled people (Longmore, 1995) in order to promote wider accessibility.

To sum up, discussions around a cripped academic knowledge production, so far, have centered harmful ableist norms in the realms of temporality and emotionality. Disability becomes a (proud) place of possibility by making their harmfulness more obvious and, thus, holds subversive potential to change orientations and priorities. Centering disability can, ultimately, make academia a more livable, caring, solidary and accessible place. Even if framed as majority issue, it remains important to question who can afford to attend to crip time in the neoliberal academy.

## 3 Ethnography

Central characteristics of ethnography are a presence in the field and an attitude of curiosity. Ethnographic research thereby focusses on implicit knowledge and forms of practice (Breidenstein et al., 2013)—or emotional and embodied forms of knowledge (Saukko, 2010), which seems fitting to the research interest described above on affective resistance to accessibility in the design of open spaces as observed by authors of Critical Access Studies (see Section 1). Fittingly, Sauerborn and Albrecht (2024) suggest ethnography as the methodology of choice to capture the observable aspects of affectivity. Ethnography grants quite a bit of methodological freedom and opportunism (Breidenstein et al., 2013)—among them a processual sharpening of the research question and methods (Flick, 2000). The most prominent ethnographic method, participant observations, produces a high quantity of complex data (Breidenstein et al., 2013), which seems equally promising for research on a topic that remains undertheorized (see Section 1).

However, ethnography suffered from the so-called crisis of representation (of *the other*) (Clifford and Marcus, 1986). This crisis challenged “an ideology claiming transparency of representation and immediacy of experience” (Clifford, 1986, 2) and instead acknowledged the co-constructed nature of cultural phenomena through practices of writing, which can ever only depict a partial truth, and does not hold authority to speak for others (Clifford, 1986; see also Said, 1978; Spivak, 1988).

“Ethnography in the service of anthropology once looked out at clearly defined others, defined as primitive, or tribal, or non-Western, or pre-literate, or nonhistorical – the list, if extended, soon becomes incoherent. Now ethnography encounters others in relation to itself, while seeing itself as other” (Clifford, 1986, 23).

In brief, ethnographic research was accused to feed into processes of othering (Harrison, 2020) – even colonization (Fuchs, 2022) – while the possibility to understand *the other* was increasingly challenged. How can ethnography be cripped then?

I argue to combine ethnographic methods with a participatory approach, where e.g., mixed-abled teams jointly or separately carry out participant observations and take individual field notes, which are then analyzed together. From a Disability Studies perspective, participation is to be understood as the cross-cutting issue in the UN Convention on the rights of people with disabilities, which has taken up demands of disability rights movements (Hirschberg and Köbsell, 2017). In ethnography, initial considerations on participatory approaches have been made under the label “collaborative ethnography” (Bettmann, 2022) and with the recommendation that its further development should be more closely linked to methodological discussions in the context of participatory research.

Furthermore, participation may serve as an epistemic moment—following feminist standpoint theories (Flick and Hoppe, 2021). An appreciation of minoritized researchers—and among them disabled researchers—as observation experts for societal relations is not new to ethnography (Breidenstein et al., 2013). Such approaches tie in well with discourses on cripping that postulate the lived experiences of disabled people can be used positively as an epistemological moment, e.g. to reveal social norms that are harmful to all members of society (see Section 2).

Ethnographic research seems attractive to co-researchers since field work is an immersive experience (Breidenstein et al., 2013), which can be more enjoyable compared to e.g. deductive analyses of transcribed interviews. Moreover, ethnographic research seems to offer grounds for participation with its opportunistic and processual character as described above. Such orientations allow for participation in the sense of negotiating and jointly deciding on research questions and methods suitable to the field and the research team—step by step. Moreover, ethnography allows for polyphony in final texts (Emerson et al., 2001; Saukko, 2010; Clifford, 1986).

However, this time- and energy-consuming research practice (Breidenstein et al., 2013) might clash with co-researches' time constraints (Hilscher, 2021; Thompson, 2021). Particularly immersive ethnographic research is described as stressful by researchers (Schmid and Eisewicht, 2022). Besides, any analysis and discussion of discrimination of one's own community requires emotional resources (Thompson, 2021). An unwillingness to meet such emotional demands should, thus, be considered (Hilscher, 2021; Thompson, 2021)—especially given the asymmetric recognition of co-researchers and researchers for their work (Russo, 2021). In this sense, a confrontation with barriers is discussed as humiliating in itself (Campbell, 2020). Therefore, a cripped practice of collaborative ethnography calls for a careful dealing with co-researchers temporal and emotional resources. Who should participate in which phases of the research process to what extent should, thus be thoroughly considered and negotiated instead of a mere declaration of symmetrical relationships between researcher and co-researchers (Flick and Herold, 2021).

Moreover, the above mentioned opportunism and freedom also means that there is no consensus on methods/techniques (Schmid and Eisewicht, 2022; Breidenstein et al., 2013). Therefore, ethnography is described as a particularly demanding research strategy, that requires researchers to be competent in various ways in order to display openness, flexibility and reflexivity (Breidenstein et al., 2013; Flick, 2000; Fuchs, 2022). Like many other qualitative methodologies, ethnography fosters a circular approach rather than a linear research process from the development of a research question, identification of a research gap based on the state of the art, design of a methodology, data collection and analysis to discussion and dissemination (Harrison, 2020). Therefore, it is more difficult to involve co-researchers only in certain aspects of the research process—if they should prefer so (Breidenstein et al., 2013). Field work usually accumulates an unsystematized corpus of field notes, which are incomprehensible to others (Emerson et al., 2001). Thus, ethnographic research might be specifically challenging to design as participatory or collaborative if the questions are asked whether co-researchers are able *and* willing to participate.

## 4 Autoethnography

One answer to ethnography's crisis of representation (of *the other*) (Clifford and Marcus, 1986) is a turn to the personal via autoethnography. Rather than hiding the researcher and author behind allegedly neutral observations and interpretations, personal experience is scrutinized as data (Anderson, 2006). Autoethnography takes serious the feminist claim that *the personal is political* while it understands both as co-constituted by the self and others (Jones and Adams, 2024). Authors seek “exposing a vulnerable self” (Ellis and Bochner, 2000, p. 739) and connect their personal experience to the wider cultural context (Ellis and Bochner, 2000). “Yet the use of personal experience alone does not make a project autoethnographic. Autoethnographers use their experience to describe, and sometimes critique, cultural beliefs, values, practices, and identities” (Jones and Adams, 2024, p. 423). That said, autoethnography combines ethnographic research – e.g. in the form of fieldwork, artifacts, field notes and *thick* descriptions—with a focus on autobiography (Jones and Adams, 2024; Ellis and Adams, 2020).

“Autoethnography is an approach to research and writing that seeks to describe and systematically analyze (graphy) personal experience (auto) in order to understand cultural experience (ethno)” (Ellis et al., 2011, n.p.). The wide range of approaches subsumed by the term autoethnography (Ellis and Bochner, 2000) can be divided into an analytic and an evocative subgenre (Anderson, 2006). The former seeks to analyze personal experience in triangulation with other data and in dialogue with sociological or cultural science theory (Anderson, 2006), whereas evocative autoethnography “repositions the reader as a coparticipant” (Ellis and Bochner, 2000, p. 744). Evocative autoethnography pursues the dialogic goal to evoke an emotional response in the sense of allowing the reader to empathize with the subject in the narrative to promote a transformative dialogue across difference. Thus, rather than narrating stories *true* to autobiographical experience, evocative autoethnography centers deeper meanings connected to these autobiographical experiences, or verisimilitude. For narrative effects, details may be changed and events collapsed (Ellis and Bochner, 2000). Effective autoethnographies, therefore, engage personal experience with theoretical frameworks or concepts, and offer complex and complicating analyses of these experiences in a narratively coherent way (Jones and Adams, 2024). Autoethnography can be described as post-qualitative research (Aberasturi-Apraiz et al., 2020) that rather seeks to change social reality to the better than describe and accurately document it (Geimer, 2011).

Given such transgression, autoethnography has attracted substantial critique—as narcissistic, quasi-therapeutic exercise beyond research (Ellis and Adams, 2020). According to Geimer (2011), qualitative research across different methodologies relies on a distinction between first- and second-order constructions in the sense of lived experiences and theoretical reconstructions of these lived experiences. In his take, autoethnography does not attempt to generate second-order constructions and, thus, forgoes indicators of rigor in qualitative research. An acknowledgment of autoethnography as qualitative methodology might, ultimately, undermine that qualitative research is taken seriously as collection and analysis of empirical data. However, a blurring of the distinction between art and (social) science is already problematized in ethnography (Clifford, 1986) as is a critique of solipsism (Fuchs, 2022).

Importantly, a focus on the personal does not have to translate to an individualistic understanding of the self. Further developments in autoethnography include relationality in the sense of collaborative witnessing and becoming part of *the other's* story or even autoethnography based on experiences by proxy such as a transgenerational transmission of trauma (Denejkina, 2017). “Perhaps autoethnography is not about the self at all; perhaps it is instead about a willful embodiment of ‘we”' (Spry, 2018, p. 628). Jones and Adams (2024, p. 421) promote a de-individualistic version of autoethnography as “becoming-with”—a relational practices that seeks to establish kinship with other people, species, environments etc. and ultimately, imagine a more just world.

Autoethnography is an approach to research that reflects a renewed attention to emotions in social and cultural science (Ellis and Bochner, 2000; Anderson, 2006; Jones and Adams, 2024; Geimer, 2011), which is particularly prominent in its evocative subgenre (Anderson, 2006) that is “showing how personal experience offers insight into the emotional, embodied, and relational aspects of culture” (Jones and Adams, 2024, p. 425). Evocative autoethnography foregrounds “what narratives do” (Ellis and Bochner, 2000, p. 746), which seems very compatible with a focus on what emotions do as promoted by theorists of affect and emotion such as Ahmed (2014) or Wetherell (2012).

Prominently, autoethnography features disability as one complex and contingent positionality influencing lived experience (Ellis and Bochner, 2000, p. 735) or (chronic) illness as “emotionally wrenching experiences” (Anderson, 2006, p. 377). Several classics of Disability Studies can be read as autoethnographic, such as Murphy's (2001) *The Body Silent* (see also Anderson, 2006) or Zola's (1982) *Missing pieces* (see also Ellis and Bochner, 2000). Besides, autoethnographic writing often centers epiphanies and existential crises that are framed as rooted in exclusion, discrimination and marginalization (Geimer, 2011). Despite such emancipatory intention, I argue that such framing can play into a *tragedy* narrative that equates disability with a pitiful functional impairment, which is further positioned as the sole explanation for the economic as well as sociocultural exclusion of disabled people. Such an individualization of the social problem disability has long been contested by activists and researchers in Disability Studies (Dobusch and Wechuli, 2020). “Given autoethnographers' critical edge, there is a tendency to tell stories about tragic events and painful experiences to promote awareness and change” (Ellis and Adams, 2020, p. 370).

Nonetheless, one key goal of autoethnography is to make research more accessible (Ellis et al., 2011; Jones and Adams, 2024) and specifically create more accessible texts (Ellis and Bochner, 2000; Ellis and Adams, 2020). More specifically, central proponents of evocative autoethnography argue that more conventional methodologies are inaccessible to minoritized researchers and readers: “For the most part, those who advocate and insist on canonical forms of doing and writing research are advocating a White, masculine, heterosexual, middle/upper-classed, Christian, able-bodied perspective” (Ellis et al., 2011, n.p.).

## 5 Concluding discussion

A perspective of cripping can counter *tragic* notions of disability and produce emancipatory knowledge for all based on disabled people's lived experiences. Using disability as an epistemological resource may change the rules of knowledge production itself, both in terms of epistemologies about disability—“what we think we know about disability, and how we know around and through it” (Johnson and McRuer, 2014a, p. 130)—and the accessibility of methodologies themselves. This paper has focused on the latter aspect in order to challenge ableism in academia, which can translate to, e.g. questioning normative time frames in data collection, analysis and dissemination as well as expectations of an implicitly able-minded performance in data collection and dissemination (see Section 2.1). Yet, issues of inaccessibility surrounding cripping itself—as an elitist and potentially exclusionary, emancipatory strategy (see Section 2)—should not be forgotten. More inclusive notions of cripping acknowledge the difficulty of proud revaluations of disability across difference and revise their terminology for the sake of accessibility.

Given the crisis of representation (of *the other*) (Clifford and Marcus, 1986), ethnography can benefit from cripping in order to develop less othering research practices. I argue that ethnography's appreciation of minoritized researchers as observation experts (Breidenstein et al., 2013) and its concession of polyphony in final texts (Emerson et al., 2001; Saukko, 2010) offer entry points for cripping. Collaborative ethnography (Bettmann, 2022) seems to be a promising extension of ethnographic approaches that might even be considered a way of cripping while ethnography's time and energy implications may conflict with temporal norms in an ableist academia, which already disadvantage disabled researchres (see Section 2.1). Similarly, the complexity of this research approach limits its accessibility (see Section 3). In other words, a cripped ethnographic design should center accessibility for a range of researchers and readers and foreground—and defend according to Harrison (2020, p. 350)—the slow modes of research the ethnographic tradition stands for: “[P]atience and attention to human complexities are under threat by assembly line modes of academic production that treat time and knowledge as commodities.” From this angle, ethnography can support claims for crip time.

Autoethnography and, particularly, its evocative subgenre developed a different answer to said crisis of representation (see Section 4). This methodology prominently features disability and focusses accessibility (Ellis and Bochner, 2000). Down to its classics, Disability Studies seem open to such an analytic use of autobiography to further an understanding of disability experiences in their cultural context. I argue that Disability Studies could largely benefit from a deeper and more systematic engagement with autoethnography. Evocative autoethnography seems to offer a particularly promising way to de-individualize (Jones and Adams, 2024) and collectivize experience. However, autoethnography risks feeding into a *tragedy* narrative of disability—not least since Disability Studies classics are framed as illness narratives (Anderson, 2006) rather than as analysis of disability as a social problem.

## Footnotes

^1^I argue for an understanding of emotion, affect and feelings as socio-cultural phenomena (Ahmed, 2014; Wetherell, 2012; Scherke, 2009) that have political functions (Ahmed, 2014) as well as social and cultural origins and impacts (Scherke, 2009; Wetherell, 2012). From this perspective, affective reactions to disability require an explanation (Scherke, 2009; Hughes, 2012).

^2^The notion of cripisthemologies also challenges epistemologies of disability as an object of knowledge (Johnson and McRuer, 2014a), which proved fruitful, for instance, for a theorization of chronic pain based on lived experiences of people living with pain (Sheppard, 2019; Patsavas, 2023).
